# Eye of the Beholder: Memory Recall Perspective Impacts Nostalgia’s Influence on Positive Affect

**DOI:** 10.3389/fpsyg.2020.572345

**Published:** 2020-09-17

**Authors:** Ross Rogers

**Affiliations:** Psychology Department, Colby College, Waterville, ME, United States

**Keywords:** nostalgia, memory, recall, perspective, positive, affect

## Abstract

Recalling memories for which one is nostalgic provides a host of psychological benefits, including promoting positive affect. The present research (*N* = 409) examined how memory recall perspective impacts this affective consequence of waxing nostalgic. Memory recall perspective research indicates that people show stronger affective engagement with memories recalled from a first person perspective (seeing the event through one’s own eyes, as one experienced it) rather than a third person perspective (seeing the event as an outside observer may have). Results indicated that when participants recalled memories from a first person perspective, those who recalled an event for which they felt nostalgic reported higher positive affect compared to their counterparts who recalled an ordinary past event. However, when participants recalled memories from a third person perspective, those who recalled an event for which they felt nostalgic reported levels of positive affect that did not differ from participants who recalled an ordinary past event. This finding suggests that, when comparing nostalgic reverie to recalling an ordinary past event, the extent to which nostalgia serves as a well-spring of positive affect is partially impacted by memory recall perspective.

Nostalgia, defined as a sentimental longing for the past, is a common emotion that serves a host of psychological functions (Routledge et al., 2013). Specifically, nostalgia boosts positive affect, increases positive self-regard, fosters feelings of social connectedness, and serves as a reservoir of meaning. Nostalgic recollections typically include the self as the central character within a meaningful social context and contain more positive rather than negative affective content (Wildschut et al., 2006). Although recent research has illuminated much about the consequences and content of nostalgic reflection, research has yet to examine the relationship between nostalgia and memory recall perspective. Memories can be recalled from first or third person perspectives (Nigro and Neisser, 1983). When engaging in first person perspective recall, individuals recall the memory from their original point of view, whereas individuals engaging in third person perspective recall see themselves in the memory, not from their original point of view, but how an observer might view the event. Autobiographical memory research suggests that the visual perspective from which memories are recalled has important consequences for how memories are experienced (Sutin and Robins, 2008). The present research provides insight into the relationship between memory recall perspective and the positive affective consequences of nostalgic reverie.

## Nostalgia

Research suggests common elements within nostalgic narratives. Wildschut et al. (2006) instructed participants to write about an event for which they felt nostalgic. Content analyses revealed that although these narratives contained aspects of both positively and negatively valanced emotions, the frequency of positive emotions was three times that of negative emotions. Additionally, nostalgic narratives overwhelmingly took on a redemptive rather than contaminating quality. That is, rarely did events turn from positive to negative, but much more often were described as shifting from negative beginnings to positive endings. Further analyses revealed that nostalgic narratives typically feature the self as the central character situated within a social context populated by significant others (e.g., family and friends). Moreover, events commemorating symbolically meaningful cultural ritual or tradition (e.g., holidays, birthdays, and graduations) are likely to be the focus of nostalgia. In sum, content analyses reveal that nostalgic narratives are predominantly positive, redemptive, self-focused, and situated in a broader context of significant others and meaningful life events.

Research has also shown that nostalgia serves a variety of vital psychological functions (Wildschut et al., 2006; Routledge et al., 2013). Nostalgia increases positive self-regard. Experiencing nostalgia, compared to control conditions, increased the accessibility of positive self-attributes and attenuated the propensity to engage in self-serving attributions following failure (Vess et al., 2012). Also, nostalgia strengthens feelings of social connectedness. Compared to control participants, nostalgic participants indicated that they felt more socially supported, loved, and protected (Zhou et al., 2008). Additional research demonstrates that individuals low (compared to high) in attachment-related avoidance (the extent to which individuals are concerned with closeness in relationships) derived more social connectedness from nostalgia and perceived a greater capacity to provide emotional support to others (Wildschut et al., 2010). Furthermore, nostalgia serves as a source of meaning. Compared to participants who thought about a positive recent past event or a positive future event, those who engaged in nostalgia displayed higher perceived presence of meaning in life and reduced search for meaning in life (Routledge et al., 2012). Additionally, research has shown that high nostalgia proneness insulates individuals from meaning threats that accompany awareness of death (Routledge et al., 2008; Juhl et al., 2010). Finally, and most germane to the present research, nostalgia increases positive affect. Participants instructed to think about a nostalgic event showed increased positive affect relative to participants who thought about an ordinary life event (Wildschut et al., 2006, 2010). In sum, abundant research indicates that nostalgia functions as an important psychological resource, providing boosts in positive self-regard, social connectedness, meaning, and positive affect.

## Memory Recall Perspective

As noted above, memories can be recalled from a first person perspective (seeing the memory through one’s own eyes, as one experienced it) or a third person perspective (seeing the memory as an observer). Research has elucidated various determinants of memory recall perspective, one of which is the individual’s emotional motivations at the time of recall (Sutin and Robins, 2008). Individuals encouraged to focus on their feelings toward a past event are more likely to recall it from a first person perspective, while individuals encouraged to focus on the concrete objective aspects of a past event are more likely to recall it from a third person perspective (Nigro and Neisser, 1983). Furthermore, research indicates that memories individuals currently associate with positive or negative affect tend to be recalled from a first person perspective, whereas memories experienced neutrally tend to be recalled from a third person perspective (D’Argembeau et al., 2003).

Research has also examined the consequences of recalling memories from a first or third person perspective. McIsaac and Eich (2002) demonstrated that memories for an experimental task recalled from a first person perspective where richer in affective reactions, physical sensations, and psychological states, while memories of the task recalled from a third person perspective included more information about spatial relations and peripheral details. Other research shows that third person perspective recall serves an emotionally distancing function, alleviating painful emotions associated with some memories (Williams and Mould, 2007). College students who recalled intrusive memories from a third person perspective reported greater detachment from and numbness toward the event compared to students who recalled similar memories from a first person perspective. However, this distancing function of third person perspective also was found for memories associated with positive emotions. Holmes et al. (2008) showed that recalling a positive event from a third person perspective resulted in less felt positive affect compared to imagining the event from a first person perspective.

Taken together, the determinants and consequences of memory recall perspective point to greater affective involvement and attachment with memories recalled from a first person perspective and more dispassionate and detached responses to memories recalled from a third person perspective. These findings suggest that visual perspective may impact the positive affective consequences of nostalgic reflection.

## The Present Research

The present research examined whether manipulating memory recall perspective impacts the usual positive affective gains of waxing nostalgic. Recalling memories from a first person perspective generally increases affective engagement with those memories compared to third person perspective recall, which is marked by distancing and detachment. I hypothesized that participants engaged in first person perspective recall who brought to mind an event for which they felt nostalgic, compared to an ordinary past event, would report higher levels of positive affect. However, this relationship would be attenuated for participants who recalled memories (nostalgic vs. ordinary past events) from a third person perspective.

## Methods

Participants completed materials online and in the order described below.

### Participants

Amazon Mechanical Turk (MTurk; Buhrmester et al., 2011) workers in the United States (*N* = 409; *M*_age_ = 33.10, *SD*_age_ = 12.15; 223 females, 108 males; six not specified; 7.1% Hispanic, 1.2% not specified; 1.2% Indian, 4.9% Asian, 1.0% American Indian/Alaska Native, 8.1% Black, 78.7% White, 3.4% Multiple, 0.2% Native Hawaiian/Pacific Islander, 1.7% Other, and 0.7% not specified) completed materials and were compensated $0.50. I aimed to sample 100 participants per condition per lab convention.

### Procedure and Materials

After providing informed consent, participants were randomly assigned to recall either an event for which they felt nostalgic or an ordinary past event and to further reflect upon the memory from either a first or third person perspective. All participants then completed a variety of measures, including a measure of affect (Watson et al., 1988), authentic living (Wood et al., 2008), self-esteem (Rosenberg, 1965), and meaning in life (Steger et al., 2006)^1^. Finally, all participants read a debriefing page and were provided information about receipt of compensation.

#### Recall Event Manipulation

After completing a filler personality measure (the 10-Item Personality Inventory, Gosling et al., 2003), participants were randomly assigned to reflect on either a nostalgic or ordinary past event. Participants assigned to the nostalgia condition read the following: “The Oxford Dictionary defines nostalgia as ‘a sentimental longing for the past’. Please bring to mind a nostalgic event in your life. Specifically, try to think of a past event that makes you feel most nostalgic.” Participants assigned to the control condition received instructions to “Please bring to mind an ordinary past event in your life. Specifically, try to think of a typical past event from your life” (adapted from Vess et al., 2012).

#### Memory Recall Perspective Manipulation

Participants were randomly assigned to further visualize the recalled event from either a first or third person perspective. Specifically, participants in the first person recall perspective condition were instructed to “Please visualize the event from the same visual perspective that you originally had, in other words, looking out at your surroundings through your own eyes.” Participants in the third person recall perspective condition were instructed to “Please visualize the event from an observer’s visual perspective, in other words, so that you can see yourself in the memory, as well as your surroundings.” Then, all participants were instructed to “Please try to make your memory image as detailed as possible. Using the space provided below, please take a few moments to write about this event” (adapted from Libby and Eibach, 2002).

#### Recall Event Manipulation Check

Participants completed a two-item recall event manipulation check (“Right now, I am feeling quite nostalgic” and “Right now, I am having nostalgic feelings”) using 1 (*strongly disagree*) to 7 (*strongly agree*) scales. Scores on the two items were combined to create a composite (*M* = 4.76, *SD* = 1.85, *r* = 0.946).

#### Affect Measure

Participants completed the 20-item state positive and negative affect schedule using 1 (*not at all*) to 5 (*extremely*) scales (PANAS; Watson et al., 1988). Example items include “Right now, I feel…excited, upset, proud, irritable.” Scores on the 10 positive affect items were combined to create a composite (*M* = 2.94, *SD* = 0.909, *α* = 0.910).

#### Demographic Items

Participants indicated their age, gender, ethnicity, race, native language, and any suspicions, thoughts, or feelings, about the study.

## Results

### Preliminary Analyses: Recall Event Manipulation Check

Scores on the recall event manipulation check composite were submitted to a 2 (Recall Event: Nostalgia vs. Ordinary) × 2 (Memory Recall Perspective: first person vs. third person) between-participants ANOVA. A significant main effect of recall event emerged. Participants who recalled an event for which they felt nostalgic reported higher felt nostalgia (*M* = 5.54, *SD* = 1.43) compared to participants who recalled an ordinary past event (*M* = 3.97, *SD* = 1.91), *F*(1,404) = 87.6, *p* < 0.001, $\eta_{p}^{2}$ = 0.178. The recall event × memory recall perspective interaction was not significant, suggesting that participants in both first and third person nostalgic memory recall conditions were experiencing nostalgia before completing the positive affect measure, *F*(1,404) = 0.138, *p* = 0.710, $\eta_{p}^{2}$ < 0.001.

### Primary Analyses: Positive Affect Measure

**Figure 1:**
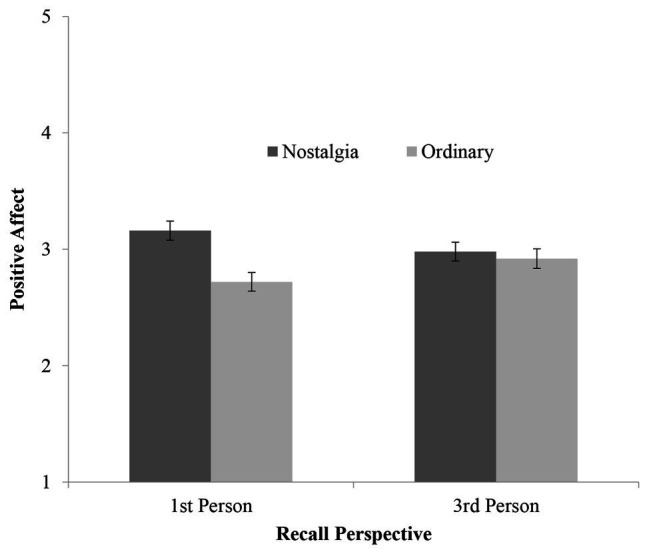
Recall event × recall perspective interaction on positive affect. Higher scores indicate higher positive affect.

Scores on the positive affect composite were subjected to a 2 (Recall Event: Nostalgia vs. Ordinary) × 2 (Memory Recall Perspective: first person vs. third person) between-participants ANOVA. A significant main effect of recall event emerged, such that participants who recalled an event for which they felt nostalgic indicated higher positive affect (*M* = 3.07, *SD* = 0.887) compared to those who recalled an ordinary past event (*M* = 2.82, *SD* = 0.917), *F*(1, 404) = 7.78, *p* = 0.006, $\eta_{p}^{2}$ = 0.019. This main effect was qualified by a significant recall event × memory recall perspective interaction, *F*(1, 404) = 4.68, *p* = 0.031, $\eta_{p}^{2}$ = 0.011 (see Figure 1). In the nostalgia condition, the difference in reported positive affect between participants in the first and third person perspective conditions was not significant, *F*(1, 404) = 2.29, *p* = 0.135, $\eta_{p}^{2}$ = 0.006, however means were consistent with the above reasoning in that participants who waxed nostalgic from a third person perspective indicated slightly lower positive affect (*M* = 2.98, *SD* = 0.916) compared to those who did so from a first person perspective (*M* = 3.16, *SD* = 0.851). Looked at differently, participants in the first person perspective condition demonstrated higher positive affect after recalling an event for which they felt nostalgic (*M* = 3.16, *SD* = 0.851) compared to those who recalled an ordinary past event (*M* = 2.72, *SD* = 0.980), *F*(1, 404) = 12.5, *p* < 0.001, $\eta_{p}^{2}$ = 0.030. However, participants in the third person perspective condition displayed no difference in positive affect after recalling either an event for which they felt nostalgic (*M* = 2.98, *SD* = 0.916) or an ordinary past event (*M* = 2.92, *SD* = 0.836), *F*(1, 404) = 0.191, *p* = 0.662, $\eta_{p}^{2}$ < 0.001.

## Discussion

Research has demonstrated nostalgia’s importance as a fount of positive affect. However, the potential influence of memory recall perspective on the positive affective outcomes of nostalgia is absent from the empirical literature. This is a notable gap because memory recall perspective impacts how memories are affectively experienced, and thus may impact nostalgic reflection’s positive affective consequences. The impetus of this research was to begin to fill this gap by examining how manipulating memory recall perspective impacts felt positive affect following nostalgic reverie or ordinary event recall.

Engagement with the emotional content of nostalgic narratives may be crucial to nostalgia’s effectiveness as a well-spring of positive affect. First person perspective recall is indicative of greater emotional engagement with a recalled event while third person perspective recall is characterized by greater detachment from recalled events. Therefore, I hypothesized and found that compared to participants who recalled an ordinary past event, those who recalled an event for which they felt nostalgic from a first person perspective indicated higher positive affect. However, recalling a nostalgic event from a third person perspective resulted in no positive affective difference compared to recalling an ordinary past event.

These initial findings may have methodological implications for nostalgia-related research. Often when the type of recalled event is manipulated in research studies, participants in the nostalgia condition are prompted to take a few moments to immerse themselves in the experience of the event for which they are nostalgic (e.g., Vess et al., 2012). Given that emotional engagement is more strongly associated with first person perspective recall (e.g., Williams and Mould, 2007; Holmes et al., 2008), such a prompt encouraging immersion in the nostalgic memory may promote recalling it from a first person perspective and thus aid in facilitating the positive affective outcomes that follow.

This research includes limitations and future directions to note. As this study is an initial examination of memory recall perspective’s impact on the positive affective consequences of nostalgia, future research is required to both corroborate and more extensively explore the relationship between memory recall perspective and nostalgic reflection. Relatedly, relevant effect sizes are small to moderate, yet are comparable to those typically observed in experimental social psychology research (Funder and Ozer, 2019). The above illuminate potential routes for further research. For example, future research should examine the extent to which, compared to other types of memory, nostalgic reflection naturally occurs from a first or third person perspective. Such research could potentially provide additional insight into nostalgia’s function as a source of positive affect. Given that memories recalled from a first person perspective tend to be more affect-laden, nostalgia’s effectiveness as a fount of positive affect may be influenced, in part, by a propensity to recall memories for which one feels nostalgic from a first person perspective.

Relatedly, research indicates that compared to non-depressed adolescents, depressed adolescents were more likely to recall autobiographical memories from a third person perspective (Kuyken and Howell, 2006). Furthermore, depressed adults remembered more positive memories from a third person perspective compared to adults who were not depressed (Legmogne et al., 2006). As research reviewed previously indicates, third person perspective recall is associated with affective distancing and detachment. Given the current findings that when recalling memories from a first person perspective, nostalgic reverie, compared to recalling an ordinary past event, resulted in higher positive affect; future research could examine how encouraging first person perspective nostalgic reflection among people living with depression may potentially produce more positive affective engagement, as well as other beneficial outcomes. Indeed, recent research explored the potential impacts of nostalgia-themed (vs. neutral) public service announcement videos about a college counseling center among a sample of college students living with depression (Hussain and Alhabash, 2020). Participants who viewed the nostalgia-themed video indicated greater positive emotion (e.g., warm and joyful) compared to those who viewed the neutral video. This effect led to more positive attitudes toward the counseling center and in turn, to greater behavioral intentions to contact the counseling center in the future.

Finally, research from a mood congruence model (MCM) perspective indicates that chronically sad people perceive mood incongruence when recalling a positive past self (Gebauer et al., 2008). This mood incongruence fosters a perception of greater temporal distance between the current (sad) self and the past (happy) self, resulting in a contrast effect and a decrease in self-esteem. However, nostalgia-related research has indicated that negative mood and loneliness can trigger nostalgia, which sometimes then serves to increase self-esteem (Wildschut et al., 2006). Perhaps these divergent findings could be reconciled by considering memory recall perspective. If, when in a negative mood, nostalgic reflection (compared to reflection on a past positive self) is engaged *via* a first person perspective, perhaps emotional connection with a “nostalgic self” fosters feelings of temporal recency despite mood incongruence. Future research should examine whether nostalgic reverie triggered by negative mood leads to increases in self-esteem, at least in part, *via* the emotional engagement characteristic of a first person perspective recall, whereas reflection on a positive past self leads to reduced self-esteem by way of the mechanisms outlined in the mood congruence model.

## Data Availability Statement

The datasets presented in this study can be found in online repositories. The names of the repository/repositories and accession number(s) can be found in the article/supplementary material.

## Ethics Statement

The studies involving human participants were reviewed and approved by Institutional Review Board, Ohio University. The patients/participants provided their written informed consent to participate in this study.

## Author Contributions

The author confirms being the sole contributor of this work and has approved it for publication.

### Conflict of Interest

The author declares that the research was conducted in the absence of any commercial or financial relationships that could be construed as a potential conflict of interest.

## Footnotes

^1^I present the results from analyses on positive affect as it was the proximal dependent variable. However, results from analyses on these measures, as well as other supplemental material, can be found here: https://osf.io/5rzwk/?view_only=fabfae927c5642628e07204b5d1dffc2
